# A scoping review of simulation models of peer review

**DOI:** 10.1007/s11192-019-03205-w

**Published:** 2019-08-19

**Authors:** Thomas Feliciani, Junwen Luo, Lai Ma, Pablo Lucas, Flaminio Squazzoni, Ana Marušić, Kalpana Shankar

**Affiliations:** 1 School of Sociology and Geary Institute for Public Policy, University College Dublin, Dublin, Ireland; 2 School of Information and Communication Studies and Geary Institute for Public Policy, University College Dublin, Dublin, Ireland; 3 Department of Social and Political Sciences, University of Milan, Milan, Italy; 4 School of Medicine, University of Split, Split, Croatia

**Keywords:** Peer review, Simulation, Agent-based modeling, Journal editing, Research funding

## Abstract

Peer review is a process used in the selection of manuscripts for journal publication and proposals for research grant funding. Though widely used, peer review is not without flaws and critics. Performing large-scale experiments to evaluate and test correctives and alternatives is difficult, if not impossible. Thus, many researchers have turned to simulation studies to overcome these difficulties. In the last 10 years this field of research has grown significantly but with only limited attempts to integrate disparate models or build on previous work. Thus, the resulting body of literature consists of a large variety of models, hinging on incompatible assumptions, which have not been compared, and whose predictions have rarely been empirically tested. This scoping review is an attempt to understand the current state of simulation studies of peer review. Based on 46 articles identified through literature searching, we develop a proposed taxonomy of model features that include model type (e.g. formal models vs. ABMs or other) and the type of modeled peer review system (e.g. peer review in grants vs. in journals or other). We classify the models by their features (including some core assumptions) to help distinguish between the modeling approaches. Finally, we summarize the models’ findings around six general themes: decision-making, matching submissions/reviewers, editorial strategies; reviewer behaviors, comparisons of alternative peer review systems, and the identification and addressing of biases. We conclude with some open challenges and promising avenues for future modeling work.

## Introduction

Peer review is a process used in the selection of manuscripts for journal publication and proposals for grant funding. It is tightly linked to the allocation of scarce resources so that those resources can be deployed to maximally foster innovation and scientific progress. Nevertheless, peer review is not a process without flaws and limitations. Researchers have identified issues related to its fairness and transparency, including conservatism and risk-taking, gender and other bias, as well as predictability, reliability, and validity issues (see, for example, Bornmann 2011; Lee et al. 2013). The importance of peer review and the awareness of its flaws have sparked scientific interest in finding the causes of (and remedies for) these issues.

Some researchers have turned to formal modeling and computer simulations to study peer review, as these methods can partially overcome the limited availability of data on internal processes at journals or funding agencies while supporting counter-factual, experimental analysis of artificial scenarios (see e.g. Squazzoni and Takács 2011). Such analyses can help test a diversity of peer review systems (real and hypothetical) while accounting for their complexity, the importance of human factors, and the high costs of interventions.

Formal models of peer review have existed since the end of the sixties (see e.g. Stinchcombe and Ofshe 1969). In the last 10 years this field of research has grown significantly but with only limited attempts to integrate disparate models or build on previous work. Thus, the resulting body of literature consists of a large variety of models, hinging on incompatible definitions, which have not been compared, and whose predictions have rarely been empirically tested.

This scoping review is an attempt to address the fragmentation in simulation studies of peer review. We do so by (1) finding existing simulation models via scoping from the two most comprehensive publication databases, Web of Science (WoS) and Scopus, as well as reference chaining; (2) reconstructing a taxonomy of the existing simulation models; and (3) providing an overview of the research questions that were investigated thus far and summarizing the models’ predictions. Our final goal is to illustrate the state of the art of simulation studies of peer review and to identify existing knowledge gaps to guide future research.

In the next sections, we will describe the advantages of formal modeling and simulation as methods to study peer review. We introduce the PRISMA-ScR protocol (Tricco et al. 2018) that we followed to perform the scoping review. We categorize the existing models based on their type, core assumptions, and research questions investigated. In doing so, we summarize the main findings from the literature, which shed light on possible ways to improve peer review. We will conclude with a discussion of the open research questions for future modeling work.

## Reviewing peer-review

Peer review is a process mainly intended to ratify the quality and validity of research. The process is largely used, for example, in reviewing manuscripts and selecting grant proposals. Generally speaking, the manuscript peer review process aims to identify the best manuscripts for publication, based on a number of evaluation criteria which include the originality of the work, appropriateness of methods, support for the reached conclusions and overall impact, as well as the fit to the focus and readership of the journal. Constructive peer review enables the authors to improve the clarity and quality of a manuscript by guiding the authors through a revision of their manuscript. Essentially, publications are expected to advance knowledge in a field and peer reviewers are gatekeepers of their respective knowledge fields. The guidelines and procedures vary depending on the publishers, journals, and editorial policies and practices.

Peer review in grant applications is in many ways different from review of manuscripts. For one, peer review in grant applications is more targeted at filtering out the worst funding applications and selecting those that are the most promising (as opposed to improving the proposals, though that is likely to happen indirectly when applicants revise their applications based on feedback). Second, reviewers may not necessarily have expertise in the specific topic of the proposal but instead may be drawn from the larger research area. Third, there is a limited timeframe for reviewers to make decisions about the distribution of grants; this time period is usually much shorter than manuscript reviews, which can occasionally take several years. And fourth, funding decisions can be based on criteria other than ‘scientific excellence’, such as the budget requested, feasibility of work plans, and strategic priorities of funding agencies. These factors/affordances/variables and the interactions between them are more complex than manuscript reviews (see, for example, Fogelholm et al. 2012; Lamont 2010; Langfeldt 2001, 2004).

Many studies have shown that peer review is not without issues and controversies. For example, research has found that inter-reviewer reliability in reviews is often not consistent (Fogelholm et al. 2012; Graves et al. 2011; Marsh et al. 2008). Low and inconsistent reliability is detrimental to the fairness, equity, transparency, and trustworthiness of peer review. There are also issues pertaining to conservatism, which prohibits potentially groundbreaking, novel, and innovative projects from being pursued and hence impedes scientific and technological process (Luukkonen 2012). Relatedly, peer review is shown to be affected by various kinds of biases, including, but not limited to, career stage, gender, language, nationality, and so on (see, for example, BioMed Central 2017; Lee et al. 2013; Marsh et al. 2008.

### Why is simulation modeling useful to address some of these problems?

The assumption underlying many studies of peer review is that we can use evidence-based methods to alleviate or solve the problems of peer review. However, studying these problems and devising solutions is not an easy task. Four factors in particular make this line of research difficult and show the advantages of simulation modeling methods. These factors are the diversity of peer review systems, their complexity, the various difficulties of accessing empirical data, and the high costs of developing and testing actual interventions.

#### Diversity

Peer review systems vary across context; for example, peer review in academic journals is implemented differently from peer review in research grants. It varies across disciplines: to give some examples, consider how careers in the humanities are evaluated differently from careers in STEM research. Evaluation criteria for peer review in conferences differ between computer science and biology. Monetary incentives to journal reviewers may be found in some fields of economics, but hardly so in other social sciences.

Peer review systems also vary within disciplines: for example, peer review may be double blind in a journal, and single blind in a different journal within the same field. Lastly, systems may also vary over time, as journals, funding agencies and conferences amend their own implementation of peer review to reflect evolving needs. This diversity makes it difficult for researchers to produce generalizable knowledge and to develop cost-effective interventions that could benefit several peer review systems.

Formal and simulation modeling tends to be most useful when the diversity of the phenomenon of interest is taken into account and we can explore the impact of different setups and interactions within the modeled system. In this case, by modeling a general peer review system, researchers can develop a minimalistic, abstract representation of a real-world peer review instance. One way of doing this is to represent only the most fundamental aspects of peer review and by doing so create a simplified model of peer review which is not necessarily context- or discipline-specific.

#### Complexity

Peer review systems hinge on non-linear interdependencies due to non-deterministic repeated interactions between heterogeneous authors/applicants, referees, editors, panel chairs and institutions. Because of these complexities, there are many facets of peer review emerging as unpredictable and/or unintended consequences over time, given typical scholarly/scientific norms and individual preferences/actions. In this sense, it is entirely possible that subtle changes in a peer review process, or in how individuals involved in it behave, could lead to non-trivial outcomes which would probably have not been anticipated. Normally, such situations would pose a problem for other research methods, but simulation methods and agent-based models have been developed specifically to facilitate answering research questions related to the study of complex systems.

#### Lack of data

The difficulty of obtaining peer review-related data is often discussed by scholars who study the topic (Lee and Moher 2017; Squazzoni et al. 2017). There are several reasons why data are scarce. On the one hand, peer review is often blind (i.e. the identity of some of the actors involved are not disclosed to some others), and the preservation of anonymity is at odds with the disclosure of data for research. On the other hand, some journals, committees and funding agencies resist external scrutiny, possibly out of concerns related to potential criticism about how peer-review has been enacted in a particular occasion.

With simulation modeling approaches, data scarcity is potentially less of a problem. When some variables cannot be quantified with empirical data, these variables can still be parameterized in a simulation model using plausible ranges of values.

#### Interventions are costly

Lastly, the study of peer review is made difficult by the challenge of developing and empirically testing interventions that contribute constructively to improving an actual peer review process while minimizing or eliminating unintended consequences. With computer simulation models we can explore the potential impact of interventions prior to any empirical implementation. Modeling potential interventions in a controlled environment can thus be a cost-effective tool to design, test, and fine-tune interventions.

## Method for the scoping review

We followed the Preferred Reporting Items for Systematic reviews and Meta-Analyses extension for Scoping Reviews (PRISMA-ScR) guidelines (Tricco et al. 2018) as they constitute an established framework for analysis of a body of literature.^1^ This scoping review encompasses all peer-reviewed publications that used computational modeling or simulation to investigate peer review processes. We adopted eligibility criteria as broad as possible so that we could capture different approaches to simulation studies across disciplines and different aspects (or types) of peer-review processes. Because this is the first literature review on the subject, the date of publication was not considered among the eligibility criteria. Our search strategies involved documents written in the English language only.

To identify the relevant sources, we followed three strategies: queries on two bibliographical databases, integration with sources from our personal knowledge, and reference chaining.

First, we queried two bibliographical databases, Web of Science (WoS) and Scopus. We used several queries, searching for one that would yield the most relevant results and the smallest proportion of irrelevant ones. Eventually we settled on a query for all indexed documents that have ever been published about “simulation” or “ABM” (or variants of those terms), and containing “peer review” in the title. The “Appendix” provides the exact search strings that we used and the complete list of references that we obtained.

The queries jointly yielded 68 unique references that were then screened by two members of our research team by reading and examining all 68 titles and abstracts and judging their relevance to the subject of our review. The decisions of inclusion or exclusion were first made independently by the two team members, and then crossed-checked for inter-reviewer reliability. The two team members agreed on the exclusion of 27 documents deemed irrelevant for this scoping review, and disagreed on 5 documents. These 5 cases were individually discussed until a decision was reached. The resulting reference list contained 36 unique documents.

In a second phase, we integrated these references with publications from our knowledge that were not captured by the queries. This step added 9 papers to our reference list (at this point, *N* = 45).

Lastly, we selected 6 of the oldest and most cited papers from our set and examined the papers that cited them to see if any were relevant to our analysis (a process often called “reference chaining”; further details about this can be found in the “Appendix”). Through this process we found an additional paper that was not already present in our reference list, bringing the final reference list to 46 documents (of which 44 are simulation studies and 2 are position papers).

It is worth noting that several articles were published by some of the authors. To minimize bias during the preparation of this scoping review we ensured that all documents were evaluated by team members other than the document’s author(s).

### Data charting

We categorized the resulting list of references through a process called “data charting” in the PRISMA-ScR guidelines. A team member (who did not author any of the references) examined all full texts and developed a charting form to guide the classification of the sources. The charting form comprised a list of attributes that we deemed relevant for organizing the literature. During the data charting, the charting form was iteratively adjusted by merging some redundant or uninformative attributes or by adding new ones. The inclusion of additional attributes allowed us to capture differences between models and approaches that we were initially unaware of.

These attributes were generally aimed at classifying the references by the key features of (and assumptions underlying) the models and by the main independent and dependent variables of interest. These attributes allowed us to distinguish among (1) the kinds of models, (2) the kinds of modeled systems, (3) the prominent model features, and (4) the research questions explored with these models. Besides classifying the references, grouping the research questions (4) is also instrumental to the presentation of the main findings from this literature.

We started by identifying the position papers. Then, for the remaining modeling papers, we identified the type of model that was being studied (1). From the more general to the more specific, we identified the formal models, the simulation models, and the agent-based models. Lastly, we identified the main groups of models: sets of papers that systematically advance our understanding of a simulation model by adding new features to previous versions of the model, or by studying the model in a different context.

A second attribute from the charting form was used to identify the modeled system (2). We distinguished between models of journal peer review from models of peer review in conferences, grants, careers, or research organizations. Then, we defined a set of attributes to identify some prominent features of the models (3). For example, we identified the models that share some key assumptions (whose relevance we will discuss later):

- The assumption that submissions have an intrinsic, objective quality level, and that it is the reviewers’ job to estimate that as accurately as possible with a review.
- The assumption that scholars have limited resources (e.g. time), and therefore face a trade-off when deciding whether to invest their resources in reviewing other scholars’ work, or in preparing their own work for submission.
- The assumption that reviewers are independent from one another when performing individual reviews.

We distinguished references based on the use of empirical data in their models: We first identified the models where at least some parameters were calibrated using real-world data. We then identified the validated models (i.e. those models whose theoretical predictions were tested against empirical data).

Lastly, we were interested in the research questions that were answered in the literature (4). Thus, we added dedicated items to our charting form that we used to classify the existing models by their research question(s) and to present their main findings. Most papers belong to one or more of these classes of models comparing:

- different aggregation rules for reviewers’ scores;
- different editorial policies;
- different reviewer behaviors;
- alternative peer-review systems;
- alternative rules for matching reviewers and submissions to be reviewed;
- models used to study the sources of bias in peer review (and which kind of bias).

## Models of peer review

As noted above, the search queries returned a raw list of 51 items from Web of Science and 47 from Scopus. Once duplicates were eliminated, we had a unique list of 68 items. The final list yielded 46 references for analysis set, 44 are modeling papers and 2 are position papers. In this section we will review the 44 modeling papers following the structure of our data charting.

### Kinds of models

Several kinds of modeling approaches were represented in our set of modeling papers. Most papers (26 cases) developed simulation experiments by means of agent-based modeling (ABM). 15 cases adopted other kinds of stochastic models, such as evolutionary models or latent Markov models. Lastly, 3 papers developed formal models of peer review that were studied analytically instead of numerically. Thus, ABMs appear to be the preferred method for simulating a peer review system.

We identified two ABMs which were particularly influential and thus earned special attention: Thurner and Hanel (2011) and Squazzoni and Gandelli (2012a, 2013). After their publication, these two models sparked their own strands of research, as they were further developed and studied in subsequent publications both by the authors and also by other scholars. Both models were originally developed to study the potential effects of different behavioral strategies that a journal reviewer could adopt. In both cases results indicate that reviewer behaviors are highly consequential: they affect both the *efficacy* of peer review (that is, how good the peer review system is at promoting the best submissions and filtering out the worst), and its *efficiency* (that is, relating to the amount of resources—e.g. time—their functioning requires).

Because several other papers built on these two models and inherited many of their characteristics, for convenience we will refer to these two ABMs as the root models, and the literature they sparked as their model group, akin to a phylogenetic tree, with branching structures. The existence of two model groups leads to a second general observation we can make about the landscape of models of peer review: it is fragmented. We found no instances of research where different existing models were combined, integrated, or tested against one another. With the exception of the two root models, most peer review models were not further developed after their first publication. This observation echoes the conclusion by Grimaldo et al. (2018, 2018) about the state of the broader literature on peer review, where fragmentation and lack of collaboration and/or knowledge sharing also prevails.

### Kinds of modeled systems

Most of the references in our set focused specifically on journal peer review (29 cases, including the two root models). Much less studied are other types of peer review systems: grants (8 cases), conferences (4 cases), career evaluation (2 cases), and evaluation of research institutions (1 case). Lastly, in 2 models peer review was defined so abstractly that it could represents all of the above types of peer review systems. This demonstrates that the systems of peer review other than peer review in journals are relatively understudied.

### Prominent model features

In this paragraph we discuss the main features, or sets of assumptions, which help classifying the existing models.

#### Intrinsic quality

First, we consider the assumption of what we call intrinsic quality. According to this assumption, what is being evaluated in the peer review (be it a paper, a grant proposal, or a CV) has an intrinsic, objectively quantified level of quality. This assumption is important because it embodies a precise perspective on what peer review is for. That is, to assume that submissions to a journal have an intrinsic quality implies that the role of the reviewer is to estimate the intrinsic quality as accurately as possible. By contrast, not assuming any intrinsic quality implies that the submission can only be evaluated subjectively: this means that, when two reviewers widely disagree on the assessment of the submission, they still may both be right.

Most of the models from our set (35 out of 44 modeling papers, including the two root models) make the assumption of intrinsic quality, explicitly or not. They define that what is being evaluated has an attribute, often named quality, expressed in one or more continuous variables. The intrinsic quality is typically assigned randomly during the initialization of the simulation. In these models, the reviewer’s assessment is calculated from the intrinsic quality: the difference between the intrinsic quality and the reviewer’s assessment is determined by some degree of evaluation error or bias.

In models without this assumption, the reviewer’s assessment is either entirely random, or based on other objective properties of what is being peer reviewed. For example, applicants can be evaluated by their research experience, or paper submissions by the seniority of the author.

#### Trade-off between resources for reviewing and preparing own submissions

Reviewers (for a journal or for a grant review panel) are usually scholars themselves. As such, their own work is typically peer reviewed by other colleagues. Some models of peer review need this dual-role of scholars to be explicitly modeled. Therefore, these models assume a realistic choice model for scholars: scholars are endowed with a finite amount of resources (e.g. time), and need to choose how much resources to invest in reviewing other scholars’ work, and how much to invest in preparing their own submissions. Models assuming this realistic choice model also assume that the quality of reviews and submissions is function of the quantity of resources invested in them.

By contrast, models without this assumption abstract from the dual-role of scholars. They propose a simpler implementation of a peer review system, where scholars (whose work is to be peer reviewed) and reviewers are two distinct populations. The quality of a submission or a review is either entirely random, or function of some other quantity (e.g. the seniority of the scholar).

The majority of the modeling papers in our set (30 out of 44) do not use this realistic choice model for scholars. This feature is the key distinction between the two groups of ABMs of peer review: whereas papers based on the root model by Squazzoni and Gandelli (2012a, 2013) assume the realistic choice model, the ones based on Thurner and Hanel (2011) do not.

#### Social influence

A manuscript, proposal or application and the information that comes with it are not the only aspects guiding a reviewer’s assessments. This is because reviewers do not do their job in complete isolation. In some peer review systems (such as review panels), the assessment is produced collectively by the reviewers through discussion. During a review panel discussion, different social processes are at play, which can determine and bias the final decision on a particular proposal (Derrick 2018; van Arensbergen et al. 2014). Similarly, in other peer review systems, reviewers come to a final assessment after having read other reviewers’ assessment, or after having read the author’s response from a previous round of review. In all of these instances, the reviewer’s final assessment results from influence dynamics, where the initial assessment can be socially influenced by repeated, complex interactions with other reviewers or with the authors, which give rise to non-linear dynamics.

The complexity inherent to social influence dynamics is an aspect of peer review, which is *absent* in most of the modeling papers in our set (41 out of 44, including the root models). In these models, for the sake of simplicity, the reviewers are assumed to act independently from their social environment, and their assessment is either randomly produced, or solely based on the properties of what they are evaluating.

Only three papers modeled at least some aspects of complex social influence dynamics in their models. Zhu et al. (2016), for instance, test the predicted effect of including two phases in the peer review process: a reviewer discussion and the author’s feedback. The reviewer discussion consists of reviewers adjusting their own score based on the scores and confidence of all reviewers; the author feedback is the opportunity for authors to improve the quality (and hopefully then the scores) of their submission. Lyon and Morreau (2018) developed an ABM to study the wisdom of crowd effects in expert panels. In Flynn and Moses (2012), social influence affects which papers will be reviewed by a reviewer: whether (and how much) the reviewer agrees with other reviewers on the assessment of a conference submission is consequential for which submissions to review next.

#### Empirical calibration and validation

Simulation models can make use of empirical data in two ways: in their calibration (when model parameters are set to an empirically-observed value) and validation (when the model predictions are empirically tested) (Hassan et al. 2010). Empirical calibration and validation of a model can be desirable for different reasons. On the one hand, calibration can reduce the parameter space that needs to be explored and can tailor the model to the social environment that is being studied. Validation, on the other hand, can provide insight into the accuracy of the model’s predictions, and thus on the goodness of our understanding of the modeled social process.

While the modeling community advocates for the use of empirical data in modeling (Hedström and Manzo 2015), few of the modeling papers on peer review use any. Out of 44 modeling papers, only 12 contained at least one empirically calibrated model parameter, and only 6 compared at least some of the model’s predictions to empirical data.

### Research questions

Here we group the 44 modeling papers by their main research questions, or aspect of peer review that was investigated. We chose the aspects that were examined in several papers and with different modeling methods or theoretical frameworks. All the following aspects have one commonality: they often emerge as crucial in determining the efficacy and efficiency of a peer review system.

#### The aggregation of reviewers’ assessments

Peer review typically relies on the reviews of two or more reviewers. In the review process, the different reviews need to be synthesized into one single score, or one single decision (e.g. to accept or to reject): hence the need for a decision rule, or some other method of aggregation of the assessments made by different reviewers into an atomic piece of information.

Four of the modeling papers tested different ways to aggregate reviewers’ assessments. Linton (2016) compares two aggregation rules: a standard averaging rule, where the final decision is the mean of the scores from all reviews, and a rule based on the Black–Scholes model (Black and Scholes 1973). The latter rule of aggregation predicts a higher acceptance rate for high-risk high-gain submissions (that is, submissions where the reviews are in disagreement). Esarey (2017) ran numerical simulations to compare other aggregation rules: acceptance upon unanimous approval by reviewers (including or excluding the editor’s opinion); acceptance upon approval by the majority of reviews (including or excluding the editor’s); unilateral editor decision based on the average review score; the effects of a desk rejection phase prior to all of the above aggregation rules. For all the above rules, the main finding is that the editor’s role and random noise are the main factors in determining the outcome of the selection process.

Righi and Takács (2017) build on the root model by Squazzoni and Gandelli (2012a, 2013). They study the alternatives that the editor of a journal has available when the reviewers disagree on a manuscript submission. Specifically, the editor can either reject the paper, accept the paper, or follow the advice of one of the reviewers. In the latter case, the editor can choose to what degree a reviewer’s reputation matters when choosing which reviewer to trust. The model shows that reviewer reputation does not contribute to better quality reviews or submissions. Surprisingly, the acceptance of controversial submissions is predicted to indirectly improve the quality of submissions: by inducing an oversupply of publishable manuscripts, this aggregation rule forces the editor to rely on the author’s reputation in order to make a decision which, in turn, incentivizes the authors to improve their reputation by investing more in submitting good quality manuscripts.

Lyon and Morreau (2018) are concerned with the composition of reviews committees, groups of reviewers who grade documents using scores and grades. Reviewers may have a different understanding (or interpretation) of scores and grades, and simulations predict that diversity in reviewers’ interpretations can foster the accuracy of the aggregated score.

#### Allocation of submissions to reviewers

In a peer review process, a key step is the selection of experts to be invited to act as reviewers. In some peer review systems, the approach is *top*-*down*: there is a person or persons (e.g. program officer, conference chair, journal editor etc.) who oversees finding and inviting a suitable potential reviewer for each given submission or proposal. In some other cases (e.g. some conferences) there is a *bidding system*. In bidding systems, a pool of potential reviewers is invited to choose among (‘bid on’) the submissions available for review, and a procedure is put in place to match submissions and reviewers based on the reviewers’ expertise and preferences.

Both the top-down and the bidding approach can be implemented in various ways. This raises the question: which approach has the most desirable outcome and under what circumstances? Some papers in our set have used simulations to answer this question.

##### Top-down allocation rules

In most of the models where the allocation is explicitly modeled, it is assumed to be random: scientists have a uniform probability to be selected as reviewers by journal editors or program officers (D’Andrea and O’Dwyer 2017; Grimaldo and Paolucci 2013; Roebber and Schultz 2011; Squazzoni et al. 2012a, 2012c, 2013). However, two papers examine alternative rules of allocation. For instance, Cook et al. (2005) test the efficacy of alternative heuristics for matching reviewers and submissions in a case: when reviewers are asked to supply their assessment in the form of an (ordinal) rank of submissions.

Cabotà et al. compare different allocation rules based on the reputation (or skill level) of authors and reviewers (Cabotà et al. 2014b). In their alternative scenarios, submissions are sent out for review to reviewers with the same reputation as the authors, to reviewers with a lower reputation, or with a higher reputation– a control treatment is examined, where reviewers are chosen randomly. The outcome variables capture the efficacy of the peer review process, its efficiency, and the inequality in the distribution of resources across scholars. Results suggest that the stronger difference between the allocation rules emerges when reviewers are systematically biased against authors with a better reputation than their own: in this case, choosing reviewers with a reputation higher than the author’s produces less biased reviews and thus improves the efficacy of peer review.

##### Allocation by bidding

Two papers focus on bidding systems. Allesina (2012) proposes an allocation system for peer review in journals which is based on a public repository of manuscripts. Authors who want to submit their own work to the public repository first have to review three other submissions of their choice. After a submission is peer reviewed, journals compete to publish it. This innovative system, it is argued, can help address some of the shortcomings of a traditional peer review system (with a top-down allocation rule).

Flynn and Moses (2012) focus on the choices available to a member of a program committee (PC) who needs to bid on the submissions that she intends to review. If the PC member wants to review the best submissions, how can she identify them and make the right bids? The authors argue how a solution to this question can be found in a search algorithm, known in computer science as the ‘ant colony optimization’ algorithm (Dorigo et al. 2006).

#### Role of the editor

In all peer review systems, there are individuals who have the final say on whether submissions, proposals or applications are to be accepted or rejected. The role of these individuals may be particularly crucial in a peer review process in two ways: through personal behavior, or through policies specifically. Their personal attitudes can directly influence which submissions to desk reject *before* the peer review process, and when to follow or disregard the reviewers’ recommendation (editor behavior). These individuals can also enact policies to change the peer review process. Examples are the selection of aggregation rules and allocation rules (which we already discussed), or the selection of how many reviewers to invite (see e.g. Kovanis et al. 2016).

The number of reviewers is one of the main manipulations in Bianchi and Squazzoni (2015, based on the root model by Squazzoni and Gandelli 2012a, 2013). Here, increasing the number of reviewers (*n* = 1 through 3) is shown to improve the accuracy of the peer review process, at the cost of increasing the amount of resources invested in the peer review.

In the model by Zhu et al. (2016), it is the program chair of a conference who can enact different policies. Various policies are explored: (a) the choice for a single blind vs. double blind review process; (b) the choice to add the chair’s own evaluation of the submission to the reviewers’; (c) to allow reviewers to be socially influenced by their peers via reviewer discussion, and (d) to allow authors to improve their submission after author feedback. By showing how all these four policies could impact the peer review process, Zhu et al. argue that the editorial choices of the program chair are of paramount importance.

In a similar vein, Wang et al. (2016) and D’Andrea and O’Dwyer (2017) extend the root model by Thurner and Hanel (2011) to include an array choices available to a journal editor (through modifying editorial policies and enacting decisions in their personal role). The editor can affect the process structure by choosing an aggregation rule, an allocation rule, and the number of reviewers to be involved in the process; she can consult a tiebreaking referee, when the initial review are in disagreement; desk-reject blatantly low quality submissions; blacklist selfish referees (referees who systematically reject submissions that they perceive as competition); and/or allow authors to revise and resubmit their manuscript.

Mrowinski et al. (2016, 2017) show how an evolutionary algorithm can be used to optimize editorial strategies by (1) minimizing the review time, and (2) keeping constant the number of reviewers involved. The model takes as input two editorial choices: how many reviewers to try to involve, and the target number of reviews. Then, based on the current state of the review process, the model can inform the editor as to how many new potential reviewers to invite, and when.

When inviting reviewers, the editor may also consider the larger review network (i.e., the network of which scholar reviews whose work). Waters et al. (2016) propose a model to study how network properties affect the efficacy of the review process. Their preliminary results identify the conditions under which clustering in the review network may have an adverse effect on the efficacy of the review process.

Lastly, Roebber and Schultz (2011) study how authors can optimally respond to editorial strategies. The model compares two strategies that scholars can follow when applying for funding: striving for quantity (submitting many proposals) or quality (submitting fewer, but of better quality). The model they develop allows to test which one is the most effective strategy depending on the editorial policy put in place by the funding program officer. Specifically, the funding officer has three choices to make: how many reviewers to invite for review; whether to base a decision on the quality of the proposal, or on the reputation of the applicant; whether or not to only fund proposal which received unanimously positive reviews. Results show that in most cases applicants are better off prioritizing quantity over quality in their proposal. There is only one case where prioritizing quality is the winning strategy: when the editorial policy requires many reviews (i.e. > 4) and the reviews must all be positive.

#### Reviewer behavior

Reviewers are the core of any peer review system. It follows that reviewer behavior and social influence play a vital role in the peer review process. Reviewers’ own attitudes and biases can come into play when reviewing a submission. Following this line of thought, Sigelman and Whicker (1987) study two dimensions of reviewers’ attitude: their severity and conventionality. Severity refers to the tendency to give generally positive (or negative) reviews; conventionality is the tendency to be harsher towards highly innovative submissions. Severity and conventionality show no effect on the effectiveness of the peer review process—a non-result that, the authors stress, may not be robust given a different parameterization of the two variables (Sigelman and Whicker 1987: 506).

The root model by Thurner and Hanel (2011) examines the effects of different reviewer strategies on the efficacy of peer review; some of these effects are examined in subsequent research (e.g. Wang et al. 2016), in some cases with adjustments (e.g. D’Andrea and O’Dwyer 2017). In particular, reviewers are considered accurate if they can correctly differentiate between good and bad quality submissions; inaccurate when their assessment is given at random; selfish if they adopt the strategic behavior of rejecting contributions of a higher quality than their own work while being accurate otherwise; altruist if they accept all contributions; and misanthropist if they reject all. Simulations consistently show that inaccurate or selfish reviewers are especially detrimental to the peer review process, as they lower the average quality of the published papers. Paolucci and Grimaldo (2014) replicate this finding and identify simulation conditions under which selfish reviewers are less detrimental, or even slightly beneficial.

The root model by Squazzoni and Gandelli (2012a, 2013) and some follow-up papers (Bianchi and Squazzoni 2015; Cabotà et al. 2013, 2014a, b) also test scenarios with varying degrees of reviewer accuracy. A control treatment where reviewers of manuscripts give accurate reviews is compared to (1) treatments where reviewers have an increasing probability of giving inaccurate reviews, (2) a treatment where reviewers are only accurate if their own manuscript was accepted (in Bianchi and Squazzoni 2015), and (3) a treatment with some conformist reviewers (i.e., reviewers who imitate other reviewers) (Cabotà et al. 2014a). These results show that, compared to the control treatment, such reviewer strategies can negatively affect both the efficacy and efficiency of peer review.

In a study looking at grant applications, Roebber and Schultz (2011) manipulate the proportion of reviewers who give an accurate vs. inaccurate (or ‘hasty’) review. Their results show that, under some conditions, inaccurate reviewers can also have a beneficial effect: they can reward applicants who apply less often, but with higher quality proposals.

Lastly, Sobkowicz (2015, 2017) proposes a simulation model of a scientific community where scholars compete for grants. The model highlights the role of reviewers’ tendency to favor proposals submitted by their close collaborators (hereafter: in-group favoritism), or to switch to more promising scientific domains. In-group favoritism in particular, even if not very prevalent, is predicted to distort the selection process through peer review.

#### Peer review systems

Many modeling papers in our set focus on peer review systems as a whole. For instance, Tan et al. (2018) focus on how a journal peer review system reacts to an external shock, such as an increase in the number of received submissions. Their model shows that the number of submissions positively correlates with journal quality, but only up until a critical saturation level. Beyond this level, more submissions result in lower journal quality.

Fang (2011) models a different kind of exogenous constraint on peer review: over-competition induced by scarcity of funding. This simulation model shows that over-competition in science can, alone, trigger a cascade where some scientific fields go extinct while a few dominant ones become monopolistic. This dynamic would not be driven by the scientific quality of the fields, nor by the goodness of the research, but solely by the self-reinforcing dynamics in the reproduction of scientists and scientific fields.

Other papers align and compare different existing peer review systems. They do so by simulating the alternative systems under the same conditions, and then measuring and comparing their efficacy and efficiency (Dignum and Dignum 2015; Zhou et al. 2016). These simulation models show how different journal peer review systems (i.e. single blind, double blind, open peer review or glance review) differ in terms of their efficacy. A more complete and recent comparison between journal peer review systems (Kovanis et al. 2016; Kovanis et al. 2017) found that while most systems are shown to outperform a conventional peer review system in at least some ways, cascade peer review emerges as the best compromise.

Furthermore, scholars have used simulation models to benchmark and develop new variants of existing peer review systems, or new systems altogether. Abramo et al. (2011), for instance, focus on national research assessments whereby a national agency use peer review to obtain a ranking of researchers or research institutions based on their research quality or productivity. Using empirical data from the Italian national assessment, the authors show that a ranking process constructed with bibliometric indicators can outperform (and be cheaper than) the traditional process based on peer review.

Grimaldo and co-authors developed two changes to a standard peer review system: reviewer accountability, also called ‘disagreement control’ (Grimaldo and Paolucci 2012, 2013; Grimaldo et al. 2012), and a reputation system (Grimaldo et al. 2018b). The idea of reviewer accountability hinges on the notion that repeated disagreement between reviewers may be a signal of poor reviews, or of selfish reviewer behavior. Thus, reviewer accountability can be implemented in traditional peer review systems (e.g. in a conference of for a journal) by banning low-quality reviewers; in other words, reviewers who consistently disagree with other reviewers are blacklisted and prevented from reviewing again for the same outlet. The second alternative, the reputation system, is explored as a viable alternative to peer review. Here, author reputation and peer review can both be used as means to filter out manuscripts which are unworthy of publication and to identify the ones which are worthy. By means of an ABM, the authors show the conditions under which reviewer accountability and reputation can be equally or even more effective and efficient than a traditional peer review system.

#### Bias in peer review

Biases can lead to scientific outputs or proposals or careers succeeding or failing on grounds unrelated to quality. For this reason, we can argue that biases are a systematic impairment of the efficacy of a peer review process.

Some of the models of peer review focus specifically on bias. Some models try to explain how biases come about, others explore the consequences of the actions of biased individuals in the peer review process, and a few try to develop solutions.

##### (a) Where bias comes from

Stinchcombe and Ofshe (1969), the oldest paper in our set, attempt to explain the emergence of evaluation bias. With a simple numerical test of a probabilistic model they show that two core conditions are enough to explain why nearly half of publishable-quality journal submissions are in fact rejected during the peer review process. The conditions are that (1) journals have a very low acceptance rate, and (2) reviewers’ estimates of the quality of a manuscript are not perfect.

Evaluation bias is even stronger when reviewers are not only somewhat inaccurate, but also biased. In a simulation study by Day (2015), the authors compare how acceptance rate varies as function of the introduction of bias against some of the submissions. Even small amounts of bias are shown to predict a large and significant detrimental effect on the success rate of applicants who are discriminated against.

Bornmann et al. (2009) investigate the origins of gender bias in PhD and postdoc fellowship applications. Expanding their previous modeling work (Bornmann et al. 2008), the authors represent the peer review of fellowship applications with a hidden Markov model, which allows to estimate the stability of the review scores through the different review stages of the application process. Their results are twofold. On the one hand, the assessment obtained during the first stage of review emerges as the most important predictor of the success of an application, and in this stage, there appears to be no gender difference. On the other hand, PhD applications show significant gender differences in the subsequent stages, where male applicants are systematically evaluated more favorably.

##### (b) The consequences of biased individuals

The model by Squazzoni and Gandelli shows the potential consequences of reviewers’ bias against submissions from authors with a different status or productivity level (Squazzoni and Gandelli 2012b, c—based on the root model by Squazzoni and Gandelli 2012a, 2013). This bias is shown to moderate the effect of reviewer’s accuracy and ultimately impact the efficacy and efficiency of the peer review process. Further work shows that evaluation bias is also affected by the interplay between the number of reviewers and their accuracy (Bianchi and Squazzoni 2015).

Other authors study the consequences of biased editors and reviewers. Particularly negative for the efficacy of peer review is the bias against highly innovative contributions, modeled as a tendency to favor conventional work and to promote a reviewer’s favored topics (Sigelman and Whicker 1987; Sobkowicz 2015). Similarly, Wang et al. (2016) explore consequences of ingroup favoritism by editors and selfish behavior by reviewers.

##### (c) A possible remedy to biased reviewers

Only three papers address potential remedies. One proposed solution is to introduce a system of reviewer accountability: simulations suggest that this can be achieved by banning reviewers who prove unreliable (Grimaldo and Paolucci 2012, 2013; Grimaldo et al. 2012). A second solution is proposed by Sobkowicz as a remedy to reviewers’ bias against highly innovative contributions, and consists in appointing an additional reviewer for submissions which prove controversial among reviewers (Sobkowicz 2015).

## Summary and discussion

Our scoping review has focused on the relatively recent but growing body of research on the use of simulation models in studying peer review. Although many other approaches have been and continue to be used to explore the mechanisms and outcomes of peer review, formal and computational modeling (and ABM in particular) offers some advantages. We have argued how simulation models can cope with the diversity and complexity of peer review systems and how they can be used to study peer review even when data are scarce and empirically testing interventions is costly or impossible.

We have (1) proposed a taxonomy of existing simulation models of peer review and (2) provided an overview of the findings from this literature branch.

The proposed taxonomy (1) was based on the main features of the models. These include the model type (e.g. formal models vs. ABMs or other) and the type of modeled peer review system (e.g. peer review in grants vs. in journals or other). We also classified the models by a set of model features (including some core assumptions) that can help distinguish between modeling approaches.

We identified a large array of research questions that were investigated with simulation models (2). The research questions can be aggregated into six general themes:

- How to aggregate the assessments by different reviewers into a single decision;
- How to best match submissions and reviewers (i.e. allocation rules);
- The role of the editor and the potential consequences of editorial strategies;
- Reviewer behavior and how different reviewing attitudes or strategies can impact peer review;
- Comparison of alternative peer review systems;
- The origins, consequences, and solutions to various biases in peer review.

### Gaps and open challenges

#### Fragmentation

The lack of collaboration and knowledge sharing in the broader literature on peer review was already discussed in previous research (Grimaldo et al. 2018a). In our scoping review we found research on peer review adopting simulation models to be just as fragmented. We found only few instances of simulation models that were further used by other authors after their first publication, and we found no attempts at comparing existing, alternative models.

Science relies on cumulative knowledge. Thus, the replication and expansion of previous research is an important target, and thus far this branch of research has largely missed it.

#### Biases

Existing models have mainly explored evaluation bias—that is, *random* errors in identifying the best proposals. The causes and consequences of systematic biases, albeit known to affect peer review (Lee et al. 2013), remain understudied or even unexplored by simulation models (see e.g. gender, ethnicity, or confirmation biases).

Furthermore, existing simulation models have mainly focused on the *consequences* of bias on the quality and good functioning of peer review. When the *causes* of bias were under scrutiny, scholars have sought (and found) them in individuals, specifically editors and reviewers. This has shifted the issue from the macro level (i.e. peer review is biased) to the micro (i.e. reviewers are biased). Put simply, modeling work shows that the reason why peer review can be biased is that individual reviewers and editors can be biased themselves.

This conclusion leads to two lines of inquiry, currently unexplored. The first one concerns other possible sources of bias: biased individuals may in fact not be the only source of bias in peer review: there may be features of the peer review process which generate institutional bias, too.

Secondly, we may argue that individual level bias is the result of a social process, too. Thus, we may ask: where does individual bias in peer review come from? Can a peer review system be designed in such a way that individual biases do not cascade into a biased peer review process?

#### Social influence

Most of the models we examined assume that reviewers act independently and that their reviews or assessment are static. However, this is not how peer review works: typically, for reviewers, the assessment of a submission results not only from reading the submission, but also from repeated interactions with the authors, editors, or the other reviewers throughout the review stages.

Scholars argue that these repeated interactions raise the possibility for reviewers to be socially influenced in their assessment and that we need to address social influence aspects if we want to fully understand peer review (Derrick 2018; van Arensbergen et al. 2014). While several social influence processes that could affect a reviewer’s evaluation of a submission have been identified and formalized in simulation models (a review can be found in Flache et al. 2017). very few simulation studies incorporated social influence aspects in the model, and none have integrated simulation models of social influence and models of peer review. Introducing social effects and attitude dynamics into simulation studies would contribute to both our understanding of peer review and of social influence processes.

#### Empirical data

Empirical data are necessary for the calibration and validation of a model to accurately simulate the intended peer review process and to test its predictions according to the interventions that have resulted from the simulation model tests. Despite calls by scholars for empirically calibrated and validated models (Hedström and Manzo 2015), and despite developments in empirical studies of peer review (see e.g. Forscher et al. 2019), few scholars in this field have pursued the empirical calibration and validation of their simulation models. As a result, the policy recommendations developed through simulation methods were never actually implemented and so their effects were never empirically tested.

This gap may be due to the limitations of quantitative data such as inappropriate and/or incomplete statistics, small or biased samples and, perhaps most noticeably to the agent-based community, the lack of access to rich qualitative data in an appropriate format to serve as input to design and implement these simulation models. Data on peer review is often qualitative, such as the content of reviews, reviewer instructions, internal guidelines regulating the process, interviews with reviewers and editors, etc. The adoption of qualitative data sources in a simulation study is potentially beneficial, but at the same time potentially difficult. For example, reviewer guidelines can be used to learn about, and thus formalize, a peer review process. Yet, to our knowledge, there are no standard protocols or best practices to guide the formalization of a process based on qualitative evidence such as reviewer guidelines. Similarly, there are no best practices on how to handle possible discrepancies between different data sources (e.g. when interviewees provide a description of a process which is in contrast with how the process is described in internal guidelines). These difficulties could be partly the reason why no modeler so far has attempted integrating diverse data sources, qualitative ones in particular. Tackling these issues presents interesting opportunities and potentially important advancements in the field of modeling and simulation.

### Conclusion

In this paper, we provide a structured review of the growing literature on the role of agent-based modeling in the study of peer review, identify its contributions to the literature and noting its gaps and opportunities for future work. Some of the following are open issues for exploration without being exhaustive: the study of biases (gender, nationality, ethnicity, and confirmation bias); cross-national studies (empirical data remains elusive and lack of comparability of models hamper such studies); simulation modeling in grant review processes; and the influence of open culture (open access publications, open peer review, and open research data) on peer review. For instance, a recent study on five Elsevier journals that recently shifted from confidential to open peer review did not find any robust negative effect of open peer review on the referee willingness to review, turnaround time, and reviewer recommendations. However, only 8% of reviewers eventually agreed on signing their reports and revealing their identity and mostly only when the report was positive (Bravo et al. 2019). Understanding long-term implications of strategic motivations and cooperation/collusion signals for the community in a simulation model without running direct experiments with scientific journals, could help inform the design of peer review systems such that biases and strategic use of this important gatekeeping function by anyone can be minimized. In this paper we argue that there are many potential areas for designing and implementing ABM, mixed methods, and collaboration for researchers/policymakers interested in the outcomes of peer review.

## Appendix

These are the search strings that we used for the two search engines:

### Web of Science (WoS)

TITLE: (“peer review”) AND TOPIC: (simulation OR “agent-based model” OR “ABM” OR “individual-based model” OR “multi-agent model” OR “multiagent model”)

### Scopus

TITLE (“peer review”) AND TITLE-ABS-KEY (simulation OR “agent-based model” OR “ABM” OR “individual-based model” OR “multi-agent model” OR “multiagent model”)

The query was run on 5 December 2018—it returned 51 results on WoS, 47 on Scopus. The following table shows the complete list of obtained references. Results from the two queries were joined to show only unique results. The references that made past the screening phase were included in the scoping review and further marked as ‘relevant’.

**Table:**

From WoS	From Scopus	Relevant	Authors	Title	Year	Source title	Volume	Issue	Page start	Page end
*	*	*	Bianchi, F., Grimaldo, F., Bravo, G., Squazzoni, F.	The peer review game: an agent-based model of scientists facing resource constraints and institutional pressures	2018	Scientometrics	116	3	1401	1420
*	*	*	Grimaldo, F., Paolucci, M., Sabater-Mir, J.	Reputation or peer review? The role of outliers	2018	Scientometrics	116	3	1421	1438
	*		Podder, V., Price, A., Sivapuram, M.S., Ronghe, A., Katta, S., Gupta, A.K., Biswas, R.	Collective conversational peer review of journal submission: a tool to integrate medical education and practice	2018	Annals of Neurosciences	25	2	112	119
	*	*	Derrick, G.	Take peer pressure out of peer review	2018	Nature	554	7690	7	
*	*		Lundeen, J.D., Warr, R.J., Cortes, C.G., Wallis, F., Coleman, J.J.	The development of a clinical peer review tool	2018	Nursing Education Perspectives	39	1	43	45
*	*	*	Righi, S., Takács, K.	The miracle of peer review and development in science: an agent-based model	2017	Scientometrics	113	1	587	607
*	*	*	Kovanis, M., Trinquart, L., Ravaud, P., Porcher, R.	Evaluating alternative systems of peer review: a large-scale agent-based modelling approach to scientific publication	2017	Scientometrics	113	1	651	671
*	*	*	Esarey, J.	Does peer review identify the best papers? A simulation study of editors, reviewers, and the scientific publication process	2017	PS—Political Science and Politics	50	4	963	969
*	*	*	Casnici, N., Grimaldo, F., Gilbert, N., Dondio, P., Squazzoni, F.	Assessing peer review by gauging the fate of rejected manuscripts: the case of the Journal of Artificial Societies and Social Simulation	2017	Scientometrics	113	1	533	546
	*	*	Mrowinski, M.J., Fronczak, P., Fronczak, A., Ausloos, M., Nedic, O.	Artificial intelligence in peer review: How can evolutionary computation support journal editors?	2017	PLoS ONE	12	9		
*	*		Cardenas, C.E., Mohamed, A.S.R., Tao, R., Wong, A.J.R., Awan, M.J., Kuruvila, S., Aristophanous, M., Gunn, G.B., Phan, J., Beadle, B.M., Frank, S.J., Garden, A.S., Morrison, W.H., Fuller, C.D., Rosenthal, D.I.	Prospective qualitative and quantitative analysis of real-time peer review quality assurance rounds incorporating direct physical examination for head and neck cancer radiation therapy	2017	International Journal of Radiation Oncology Biology Physics	98	3	532	540
*	*		Mitchell, J.D., Chesnut, T.J., Eastham, D.V., Demandante, C.N., Hoopes, D.J.	Detailed prospective peer review in a community radiation oncology clinic	2017	Practical Radiation Oncology	7	1	50	56
*		*	Pier, Elizabeth L.; Raclaw, Joshua; Kaatz, Anna; Brauer, Markus; Carnes, Molly; Nathan, Mitchell J.; Ford, Cecilia E.	Your comments are meaner than your score’: score calibration talk influences intra-and inter-panel variability during scientific grant peer review	2017	Research Evaluation	26	1	1	14
*			[Anonymous]	Peer review report 1 on Impact analysis of satellite rainfall products on flow simulations in the Magdalena River Basin, Colombia	2017	Journal of Hydrology-Regional Studies	9		112	113
*			[Anonymous]	Peer review report 2 on Impact analysis of satellite rainfall products on flow simulations in the Magdalena River Basin, Colombia	2017	Journal OF Hydrology-Regional studieS	9		114	114
*			Rodrigues, Lineu	Peer review report 1 on hydrological simulation in a basin of typical tropical climate and soil using the SWAT Model Part I: calibration and validation tests	2017	Journal of Hydrology-Regional Studies	9		115	115
*			Rocha, Felizardo Adenilson	Peer review report 2 on hydrological simulation in a basin of typical tropical climate and soil using the SWAT model Part I: calibration and validation tests	2017	Journal of Hydrology-Regional Studies	9		116	116
*	*	*	Wang, W., Kong, X., Zhang, J., Chen, Z., Xia, F., Wang, X.	Editorial behaviors in peer review	2016	SpringerPlus	5	1	1	11
*	*	*	Zhu, J., Fung, G., Wong, W.H., Li, Z., Xu, C.	Evaluating the pros and cons of different peer review policies via simulation	2016	Science and Engineering Ethics	22	4	1073	1094
*	*	*	Mrowinski, M.J., Fronczak, A., Fronczak, P., Nedic, O., Ausloos, M.	Review time in peer review: quantitative analysis and modelling of editorial workflows	2016	Scientometrics	107	1	271	286
*	*	*	Kovanis, M., Porcher, R., Ravaud, P., Trinquart, L.	Complex systems approach to scientific publication and peer-review system: development of an agent-based model calibrated with empirical journal data	2016	Scientometrics	106	2	695	715
	*	*	Stevens, S., Waters, A., Babik, D., Tinapple, D.	Efficacy of peer review network structures: the effects of reciprocity and clustering	2016	2016 International Conference on Information Systems, ICIS 2016				
*			Rocha, Felizardo Adenilson	Peer review report 1 on hydrological simulation in a basin of typical tropical climate and soil using the SWAT model part II: simulation of hydrological variables and soil use scenarios	2016	Journal of Hydrology-Regional Studies	5		51	51
*			Rodrigues, Lineu	Peer review report 2 on hydrological simulation in a basin of typical tropical climate and soil using the SWAT model Part II: simulation of hydrological variables and soil use scenarios	2016	Journal of Hydrology-Regional Studies	5		52	52
*			[Anonymous]	Peer review report 2 on the effectiveness of google glass as a vital signs monitor in surgery: a simulation study	2016	International Journal of Surgery	25		404	404
*			[Anonymous]	Peer review report 3 on the effectiveness of google glass as a vital signs monitor in surgery: a simulation study	2016	International Journal of Surgery	25		405	405
*			Ameh, Emmanuel A.	Peer review report 1 on the effectiveness of google glass as a vital signs monitor in surgery: a simulation study	2016	International Journal of Surgery	25		415	415
*		*	Zhou, Jian; Cai, Ning; Li, Yanjun	Analysis of peer review system based on fewness distribution function	2016	Proceedings of the 2016 6th International Conference on Management, Education, Information and Control (MEICI 2016)	135		1133	1136
*	*	*	Bianchi, F., Squazzoni, F.	Is three better than one? simulating the effect of reviewer selection and behavior on the quality and efficiency of peer review	2015	Proceedings—Winter Simulation Conference	2016-February		4081	4089
*	*	*	Day, T.E.	The big consequences of small biases: A simulation of peer review	2015	Research Policy	44	6	1266	1270
*	*	*	Sobkowicz, P.	Innovation suppression and clique evolution in peer-review-based, competitive research funding systems: An agent-based model	2015	JASSS	18	2	1	23
*			[Anonymous]	Peer review report 2 on Simulation of river flow in the Thames over 120 years: evidence of change in rainfall-runoff response?	2015	Journal of Hydrology-Regional Studies	3		52	53
*			[Anonymous]	Peer review report 1 on Simulation of river flow in the Thames over 120 years: evidence of change in rainfall-runoff response?	2015	Journal of Hydrology-Regional Studies	3		54	56
*			Saaltink, Remon	Peer review report 1 on A simulation based approach to quantify the difference between event-based and routine water quality monitoring schemes	2015	Journal of Hydrology-Regional studies	3		104	106
*			Bartley, Rebecca	Peer review report 2 on a simulation based approach to quantify the difference between event-based and routine water quality monitoring schemes	2015	Journal of Hydrology-Regional Studies	3		109	109
*			Villen-Guzman, M.; Garcia-Rubio, A.; Paz-Garcia, J. M.; Diaz-Morilla, A.; Sanchez-Molina, M.; Vereda-Alonso, C.; Gomez-Lahoz, C.	Comparison of students’ satisfaction with the peer-review workshops between two academic years in the subject simulation and optimization	2015	ICERI2015: 8th International Conference of Education, Research and Innovation			7654	7659
*		*	Dignum, Virginia; Dignum, Frank	Exploring social practices of peer-review in an agent-based simulation: the COST action PEERE	2015	21st International Congress on Modelling and Simulation (MODSIM2015)			1902	1908
*	*		Haier, R.J., Karama, S., Colom, R., Jung, R., Johnson, W.	A comment on “fractionating intelligence” and the peer review process	2014	Intelligence	46		323	332
	*	*	Cabotà, J.B., Grimaldo, F., Squazzoni, F.	Do editors have a silver bullet? an agent-based model of peer review	2014	Proceedings—28th European Conference on Modelling and Simulation, ECMS 2014			725	731
*	*	*	Paolucci, M., Grimaldo, F.	Mechanism change in a simulation of peer review: from junk support to elitism	2014	Scientometrics	99	3	663	688
*	*	*	Cabotà, J.B., Grimaldo, F., Squazzoni, F.	When competition is pushed too hard. An agent-based model of strategic behaviour of referees in peer review	2013	Proceedings—27th European Conference on Modelling and Simulation, ECMS 2013			881	887
*	*	*	Squazzoni, F., Gandelli, C.	Opening the black-box of peer review: an agent-based model of scientist behaviour	2013	JASSS	16	2		
*	*	*	Grimaldo, F., Paolucci, M.	A simulation of disagreement for control of rational cheating in peer review	2013	Advances in Complex Systems	16	7		
*	*	*	Squazzoni, F., Gandelli, C.	Peer review under the microscope. An agent-based model of scientific collaboration	2012	Proceedings—Winter Simulation Conference				
*	*	*	Flynn, M., Moses, M.	Improving peer review with ACORN: ACO algorithm for reviewer’s network	2012	Lecture Notes in Computer Science (including subseries Lecture Notes in Artificial Intelligence and Lecture Notes in Bioinformatics)	7461 LNCS		260	267
*	*		Yankulov, K., Couto, R.	Peer review in class: metrics and variations in a senior course	2012	Biochemistry and Molecular Biology Education	40	3	161	168
*	*	*	Squazzoni, F., Gandelli, C.	Saint Matthew strikes again: an agent-based model of peer review and the scientific community structure	2012	Journal of Informetrics	6	2	265	275
	*	*	Grimaldo, F., Paolucci, M., Conte, R.	Agent simulation of peer review: the PR-1 model	2012	Lecture Notes in Computer Science (including subseries Lecture Notes in Artificial Intelligence and Lecture Notes in Bioinformatics)	7124 LNAI		1	14
*	*	*	Liu, X.Z., Fang, H.	Peer review and over-competitive research funding fostering mainstream opinion to monopoly. Part II	2012	Scientometrics	90	2	607	616
	*		Kapoor, R., Kapur, P., Kumar, S.A., Alex, D., Ranka, S., Palta, J.	SU‐E‐T‐211: peer review system for ensuring quality of radiation therapy treatments	2012	Medical Physics	39	6	3751	3752
*			Smith, Maxwell L.; Raab, Stephen S.	Directed peer review in surgical pathology	2012	Advances in Anatomic Pathology	19	5	331	337
*		*	Squazzoni, Flaminio; Gandelli, Claudio	Opening the black-box of referee behaviour. an agent-based model of peer review	2012	Proceedings 26th European Conference on Modelling and Simulation ECMS 2012			647	653
*		*	Grimaldo, Francisco; Paolucci, Mario	A simulation of disagreement for control of rational cheating in peer review	2012	Proceedings 26th European Conference on Modelling and Simulation ECMS 2012			676	+
*	*	*	Thurner, S., Hanel, R.	Peer-review in a world with rational scientists: toward selection of the average	2011	European Physical Journal B	84	4	707	711
	*	*	Abramo, G., D’Angelo, C.A., Costa, F.D.	National research assessment exercises: a comparison of peer review and bibliometrics rankings	2011	Scientometrics	89	3	929	941
*	*	*	Roebber, P.J., Schultz, D.M.	Peer review, program officers and science funding	2011	PLoS ONE	6	4		
*	*	*	Squazzoni, F., Takács, K.	Social simulation that ‘peers into peer review’	2011	JASSS	14	4		
*	*	*	Fang, H.	Peer review and over-competitive research funding fostering mainstream opinion to monopoly	2011	Scientometrics	87	2	293	301
	*		Sheu, Y.R., Feder, E., Balsim, I., Levin, V.F., Bleicher, A.G., Branstetter IV, B.F.	Optimizing radiology peer review: a mathematical model for selecting future cases based on prior errors	2010	Journal of the American College of Radiology	7	6	431	438
	*		Pine, M., Fry, D.E.	Linking processes and outcomes to improve surgical performance: A new approach to morbidity and mortality peer review	2006	American Surgeon	72	11	1115	1119
	*		Clifford, R.M., Plumridge, R.J.	Developing key competencies for a clinical pharmacy education and peer review program	2003	Journal of Pharmacy Practice and Research	33	3	208	211
	*		Mercer, M., Fitzpatrick, M.	Technical highlights of EPA peer review of NAPL web site	2002	Proceedings of the Third International Conference on Remediation of Chlorinated and Recalcitrant Compounds			311	318
	*		Weatherley, J., Sumner, T., Khoo, M., Wright, M., Hoffmann, M.	Partnership reviewing: a cooperative approach for peer review of complex educational resources	2002	Proceedings of the ACM International Conference on Digital Libraries			106	114
	*		D’Astous, P., Robillard, P.N.	Empirical study of exchange patterns during software peer review meetings	2002	Information and Software Technology	44	11	639	648
	*		Schonemann, P.H.	Better never than late: peer review and the preservation of prejudice	2001	Ethical Human Sciences and Services	3	1	7	21
	*		Bodner, S., Paine, C.	When peer review fails: problems with a giant laser project	2000	Nature	407	6801	129	130
	*		d’Astous, Patrick, Robillard, Pierre N.	Characterizing implicit information during peer review meetings	2000	Proceedings—International Conference on Software Engineering			460	466
*		*	SIGELMAN, L; WHICKER, ML	Some implications of bias in peer-review—a simulation-based analysis	1987	Social Science Quarterly	68	3	494	509

### Data charting

The following table shows the outcome of the data charting performed on the 46 relevant references.

Note: the table also shows references that were added from our personal knowledge or found via reference chaining (‘source’ is marked as ‘personal knowledge’ or ‘citation chaining’, respectively).

**Table:**

	Source		Model taxonomy							Research questions					
	Source	Seed for citation chaining	Kind of simulation model	Kind of peer review	Intrinsic quality	Trade-off authoring/reviewing	Social influence	Calibration	Validation	On score aggregation	On matching submissions/reviewers	On editor’s role	On reviewer behavior	On peer review systems	On bias
Lyon and Morreau (2018)	Personal knowledge		Other simulation model	Any	*		*	*		*					
Bianchi et al. (2018)	WoS and Scopus		ABM	Journal	*	*									*
Grimaldo, Marušic, et al. (2018a)	WoS and Scopus		ABM	Journal	*			*						*	
Derrick (2018)	Scopus		NA—position paper	Career								*			*
Tan et al. (2018)	Citation chaining		other simulation model	Journal	*									*	
Righi and Takács, (2017)	WoS and Scopus		ABM	Journal	*	*				*		*			*
Kovanis et al. (2017)	WoS and Scopus		ABM	Journal	*	*								*	
Esarey (2017)	WoS and Scopus		Other simulation model	Journal	*					*		*			
Mrowinski et al. (2017)	Scopus		Evolutionary algorithm	Journal								*			
Sobkowicz (2017)	Personal knowledge		ABM	Grant				*	*						
D’Andrea and O’Dwyer (2017)	Personal knowledge		ABM	Journal	*							*	*		*
Wang et al. (2016)	WoS and Scopus		ABM	Journal	*					*		*	*		
Zhu et al. (2016)	WoS and Scopus		ABM	Journal	*		*	*				*	*	*	*
Mrowinski et al. (2016)	WoS and Scopus		Other simulation model	Journal				*							
Kovanis et al. (2016)	WoS and Scopus		ABM	Journal	*			*						*	
Waters et al. (2016)	Scopus		Other simulation model	Career	*										
Zhou et al. (2016)	WoS		formal model	Journal								*		*	
Linton (2016)	Personal knowledge		formal model	Any						*		*			
Bianchi and Squazzoni (2015)	WoS and Scopus		ABM	Journal	*	*						*	*		*
Day (2015)	WoS and Scopus		Other simulation model	Grant	*			*							*
Sobkowicz (2015)	WoS and Scopus		ABM	Grant	*								*		*
Dignum and Dignum (2015)	WoS		Other simulation model	Journal	*									*	
Cabotà et al. (2014a)	Personal knowledge		ABM	Journal	*	*							*		
Cabotà et al. (2014b)	Scopus		ABM	Journal	*	*					*	*	*		*
Paolucci and Grimaldo (2014)	WoS and Scopus		ABM	Conference	*								*		
Cabotà et al. (2013)	WoS and Scopus		ABM	Journal	*	*							*		*
Squazzoni and Gandelli (2013)	WoS and Scopus		ABM	Journal	*	*							*		
Grimaldo and Paolucci (2013)	WoS and Scopus		ABM	Conference	*	*							*	*	
Squazzoni and Gandelli (2012b)	WoS and Scopus		ABM	Journal	*	*							*		
Flynn and Moses (2012)	WoS and Scopus		ABM	Journal	*		*				*	*			
Squazzoni and Gandelli (2012c)	WoS and Scopus		ABM	Journal	*	*							*		*
Grimaldo et al. (2012)	Scopus		ABM	Conference	*									*	
Liu and Fang (2012)	WoS and Scopus		other simulation model	Grant	*			*	*						
Squazzoni and Gandelli (2012a)	WoS	*	ABM	Journal	*	*							*		
Grimaldo and Paolucci (2012)	WoS		ABM	Conference	*	*							*	*	
Allesina (2012)	Personal knowledge		ABM	Journal	*	*					*	*			
Thurner and Hanel (2011)	WoS and Scopus	*	ABM	Journal	*								*		
Abramo et al. (2011)	Scopus	*	Other simulation model	Institutions				*	*					*	
Roebber and Schultz (2011)	WoS and Scopus		ABM	Grant	*			*				*	*		
Squazzoni and Takács (2011)	WoS and Scopus	*	NA—position paper	Journal								*	*		*
Fang (2011)	WoS and Scopus	*	Other simulation model	Grant	*			*	*					*	
Bornmann et al. (2009)	Personal knowledge		Markov process	Grant					*						*
Bornmann et al. (2008)	Personal knowledge		Markov process	Grant					*						
Cook et al. (2005)	Personal knowledge		Other simulation model	Journal							*	*			
Sigelman and Whicker (1987)	WoS	*	Other simulation model	Journal	*								*		*
Stinchcombe and Ofshe (1969)	Personal knowledge		Formal model	Journal	*			*							*

## Footnotes

1 It is worth noting that PRISMA-ScR guidelines are tailored for scoping reviews with a registered review protocol and recommend registration. However, a review protocol was not necessary in our case.
